# The antitumor action of endocannabinoids in the tumor microenvironment of glioblastoma

**DOI:** 10.3389/fphar.2024.1395156

**Published:** 2024-04-24

**Authors:** Yi Tang, Maoru Wang, Jiangping Yu, Guangyao Lv, Yu Wang, Bin Yu

**Affiliations:** ^1^ Department of Pharmacy, Sichuan Cancer Center, Sichuan Cancer Hospital and Institute, Affiliate Cancer Hospital of University of Electronic Science and Technology of China (UESTC), Chengdu, China; ^2^ Drug Dispensing Department, Sichuan Mental Health Center, The Third Hospital of Mianyang, Mianyang, China; ^3^ Department of Pharmacy, Mianyang Central Hospital, School of Medicine, University of Electronic Science and Technology of China, Mianyang, China; ^4^ Key Laboratory of Molecular Pharmacology and Drug Evaluation, School of Pharmacy, Collaborative Innovation Center of Advanced Drug Delivery System and Biotech Drugs in Universities of Shandong, Yantai University, Yantai, China

**Keywords:** endocannabinoids, glioblastoma, tumor microenvironment, CB1R, CB2R

## Abstract

Approximately 80% of all malignant brain tumors are gliomas, which are primary brain tumors. The most prevalent subtype of glioma, glioblastoma multiforme (GBM), is also the most deadly. Chemotherapy, immunotherapy, surgery, and conventional pharmacotherapy are currently available therapeutic options for GBM; unfortunately, these approaches only prolong the patient’s life by 5 years at most. Despite numerous intensive therapeutic options, GBM is considered incurable. Accumulating preclinical data indicate that overt antitumoral effects can be induced by pharmacologically activating endocannabinoid receptors on glioma cells by modifying important intracellular signaling cascades. The complex mechanism underlying the endocannabinoid receptor-evoked antitumoral activity in experimental models of glioma may inhibit the ability of cancer cells to invade, proliferate, and exhibit stem cell-like characteristics, along with altering other aspects of the complex tumor microenvironment. The exact biological function of the endocannabinoid system in the development and spread of gliomas, however, is remains unclear and appears to rely heavily on context. Previous studies have revealed that endocannabinoid receptors are present in the tumor microenvironment, suggesting that these receptors could be novel targets for the treatment of GBM. Additionally, endocannabinoids have demonstrated anticancer effects through signaling pathways linked to the classic features of cancer. Thus, the pharmacology of endocannabinoids in the glioblastoma microenvironment is the main topic of this review, which may promote the development of future GBM therapies.

## 1 Introduction

Glioma is one of the most difficult and resistant to treatment malignancies. Approximately 80% of all malignant brain tumors are gliomas (Louis et al., 2016). According to the World Health Organization (WHO), gliomas are classified as grade I (benign) through IV (malignant). Glioblastoma multiforme (GBM) is the most prevalent primary brain tumor in adults, with an annual incidence of 3.19 new cases per 100,000 people. GBM is also the most fatal primary brain cancer, with a 2-year survival rate of 26%–33%, a 5-year survival rate of 4%–5% (Batash et al., 2017), and a mere 15-month median survival time. Notably, GBM patients often have a poor prognosis despite the use of rigorous, multimodal therapy, including immunotherapy, chemotherapy, and radiation therapy, in addition to surgical excision (Batash et al., 2017).

The median survival rate for individuals with GBM is less than 2 years after diagnosis and has not significantly improved in recent decades, despite the development of extensive multidisciplinary therapies (McCutcheon and Preul, 2021). Standard GBM treatment strategies include radiotherapy and chemotherapy after extensive surgical resection (McCutcheon and Preul, 2021). Treatments for recurrent or progressing GBM include surgery, reirradiation, systemic therapies, combination modality therapy, and supportive care (Fernandes et al., 2017). Because many cell subpopulations, including glioma stem cells (GSCs), are present within the tumor mass, GBMs typically exhibit considerable pathological, genetic and structural heterogeneity. For example, GSCs are a small subpopulation of cancer cells that are both pluripotent and self-renewing (Prager et al., 2020; Biserova et al., 2021). GSCs continue to proliferate unchecked, which facilitates the growth and recurrence of tumors. Fast-dividing GSC progenitor cells are necessary for rapid tumor growth; tumor recurrence is frequently caused by poor mitotic activity of GSCs. These cells are shielded from numerous therapies that actively target dividing cells because of their low mitotic activity. GSCs can therefore endure these therapies and lead to recurrence (Biserova et al., 2021). To improve the prognosis and quality of life of GBM patients, efficacious medicines targeting both GBM cells and GSCs are desperately needed.

The largest superfamily of biological receptors, G protein-coupled receptors (GPCRs), has drawn much interest recently among the many signal transduction platforms that are impacted in glioblastoma cells (Cherry and Stella, 2014; Byrne et al., 2021). Glioma cells express endocannabinoid-sensing GPCRs (type-1 cannabinoid receptor, CB1R; and type-2 cannabinoid receptor, CB2R), which are pharmacologically activated to target various cancer hallmarks, including angiogenesis, proliferation, resistance to programmed cell death, invasiveness, and metastasis (Dumitru et al., 2018; Ellert-Miklaszewska et al., 2020). In addition, compounds called endocannabinoids are produced when arachidonic acid is broken down. 2-Arachidonoylglycerol (2-AG) and anandamide (AEA) are the first two endocannabinoids. 2-AG and AEA are essential for the growth, control, migration, and maturity of neural brain cells. Neurons produce both AEA and 2-AG, which regulate the release of glutamate and c-aminobutyric acid (Citti et al., 2018).

GBM is particularly resistant to growth factors such as vascular endothelial growth factor (VEGF) and tyrosine kinase receptors such as phosphatidylinositol 3-kinase (PI3K), as well as current anticancer medicines (Dang et al., 2009; Chowdhury et al., 2011). Thus, interest in alternative therapeutic methods for GBM has increased. At this point, endocannabinoids might be a good alternative treatment for GBM. To provide a promising strategy for the future treatment of this deadly disease, current developments in the dysregulation of the endocannabinoid system (ECS) in GBM and the potential of the endocannabinoids as a therapeutic target is examined in this review.

## 2 Expression of the endocannabinoid system in glioma

ECS, which is a crucial neuromodulatory network that regulates a wide range of biological processes, is composed of endocannabinoids, their receptors, and the proteins involved in their synthesis, transport, degradation, and bioconversion. Currently, the two main cannabinoid-specific receptors, CB1R and CB2R, have been isolated from mammalian tissues. In the central and peripheral nervous systems, most of the actions of cannabinoids are dependent on activating CB1Rs, which are mostly found on neurons. CB2Rs are more prevalent in immune cells and also be found in other types of cells, such as cancer cells. The enzymes N-acyl-phosphatidylethanolamine-phospholipase D (NAPE-PLD) and diacylglycerol lipase α/β (DAGLα/β; DAGLα accounts for most 2-AG production in the adult brain) are the main producers of the cannabinoid receptor ligands AEA and 2-AG from membrane lipids. The enzymes monoacylglycerol lipase (MAGL) and fatty acid amide hydrolase (FAAH) are primarily responsible for deactivating 2-AG and AEA, respectively (Katona and Freund, 2008; Castillo et al., 2012).

Glial cells have long been known to have a functioning CB1R and CB2R, with the former being more prevalent in neuroglia and the latter in microglia (Stella, 2010). Similarly, CB1R and CB2R mRNA and protein are expressed at detectable levels in a variety of glioma cell lines (Vaccani et al., 2005; Lorente et al., 2011). Overall, the upregulation of CB2R expression in high-grade glioma samples is the most consistent observation across these studies (De Jesús et al., 2010; Wu et al., 2012; Hashemi et al., 2020). Although tumor cells also express this receptor, CB2R is found mostly in infiltrating immune cells and blood vessel endothelial cells (Held-Feindt et al., 2006; Hashemi et al., 2020). Conversely, there have been reports of increases, decreases, or no changes in CB1R expression in biopsies of high-grade gliomas. Two investigations indicated that CB1R expression was upregulated compared to that in nearby, nontumoral tissue (Wu et al., 2012; Hashemi et al., 2020), and plasma membrane preparations, rather than entire tissue homogenates, were used in a publication that showed a lower CB1R density (De Jesús et al., 2010).

CB1R appears to be primarily expressed on glioma cells, in contrast to CB2R, as indicated by its colocalization with the astrocytic marker glial fibrillary acidic protein (GFAP) (Wu et al., 2012; Hashemi et al., 2020). *In vitro* and *in vivo*, gliomas express the transient receptor potential vanilloid 1 (TRPV1) receptor in addition to CB1R and CB2R. Moreover, in contrast to findings in Δ9-THC (Galve-Roperh et al., 2000; Sánchez et al., 2001), glioma cell death caused by AEA is mediated by TRPV1 rather than CB1R/CB2R (Contassot et al., 2004). Additionally, TRPV2 receptor expression is much greater in benign astrocyte tissue, but this expression gradually decreases as tumor histological grade increases in glioma tissue (Nabissi et al., 2010). Reduced levels of AEA were discovered in meningioma samples and in a single glioblastoma biopsy according to another investigation (Maccarrone et al., 2001). As AEA levels in *postmortem*, anoxic brains increase with time (Schmid et al., 1995), these differences could be the result of various sample handling procedures used in different studies. To our knowledge, one study has examined the expression of enzymes involved in the metabolism of 2-AG. The DAGLα levels in the glioblastoma samples were found to be similar to those in the matched controls, whereas MAGL expression was much lower. These findings were consistent with the increased levels of 2-AG (Wu et al., 2012). This result is consistent with that of another study that reported higher levels of 2-monoacylglycerols in glioma tissues, even though there was no discernible substantial increase in 2-AG in this study (Petersen et al., 2005). Taken together, these findings imply that the 2-AG-CB2R signaling axis may be hyperactive in human gliomas; nevertheless, data related to most system components are limited and occasionally contradictory. Therefore, additional studies are needed to determine which specific tumor cell types express various ECS components and how the dynamic regulation of these proteins and lipids changes as the disease progresses toward malignancy.

## 3 GBM tumor microenvironment

**FIGURE 1:**
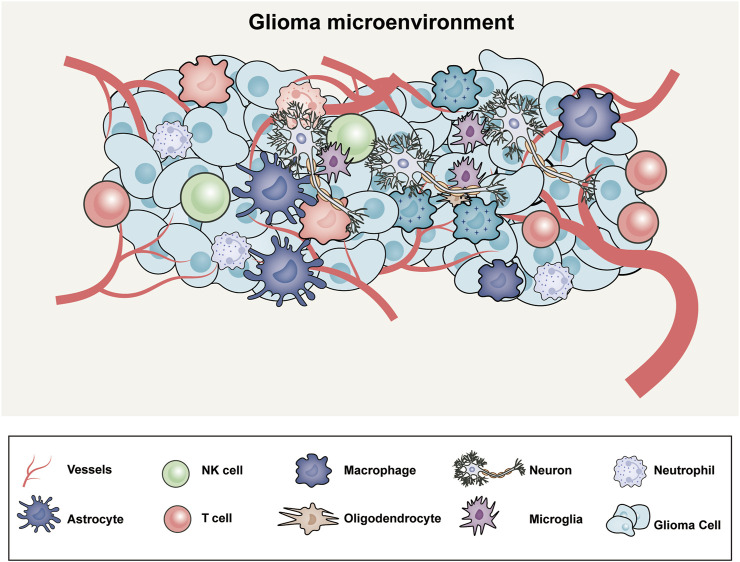
The microenvironment of glioblastoma includes an intricate web of abnormal blood vessels, tumor cells, extracellular matrix, neurons, and glial and immune modulators.

The tumor microenvironment (TME) has been neglected and undervalued in the development of therapeutic approaches, although the genetics of GBM have been the subject of substantial research. The TME, which is composed of elements of the organismal milieu and the tumor niche, regulates the growth and invasion of GBM (Bikfalvi et al., 2023). The extracellular matrix (ECM) surrounds each of these cell types and soluble factors that affect tumor growth, immune evasion, angiogenesis, invasion, and drug resistance. While glioma microenvironment includes macrophages derived from bone marrow, myeloid cells (resident microglia), tissue-resident cells (astrocytes, oligodendrocytes and neurons), NK cells, neutrophil, T cells and glioma cells (Figure 1). Because of the different genetic abnormalities and chromosomal alterations that impair the normal flow of cell signaling via growth factors or cytokines, cell-to-cell signaling is essential for the growth of tumors. Within a tumor, this signaling occurs between cells that are hypoxic and normoxic or between nearby and far-off cells in the TME. The “go or grow” behavior of glioma cells, which determines whether they migrate or proliferate, may be impacted by numerous environmental stimuli. The TME was mathematically modeled using a reaction‒diffusion equation to understand and simulate how various components involved in tumor growth interact and spread. A mathematical model and a transwell experiment in which microglia secrete transforming growth factor β to promote glioma cells in the laboratory were used to validate the model. By creating these models, investigating a variety of speculative scenarios and forecasting how a tumor would behave under various circumstances will be possible; these models will increase the amount of experimental data and emphasize the importance of the tumor microenvironment (Kim et al., 2017).

Systemic elements that influence tumor development and response to therapy, such as the blood–brain barrier permeability, hormonal conditions, metabolic states, and the microbiome, are referred to as the GBM tumor macroenvironment. Tumors release substances that cause the host to create an environment in which different distant compartments beyond the local tumor location communicate with one another. When tumors are present, this milieu can lead to systemic changes, including modifications in bone marrow function, specifically in myelopoiesis. Normal myeloid cell differentiation in cancer is also redirected from its intrinsic pathway of terminal differentiation to mature myeloid cells, including dendritic cells, macrophages, and granulocytes, toward a pathway activated by signals derived from the TME that generates pathologically activated immature and immunosuppressive cells. This process is in contrast to emergency myelopoiesis, which is induced by acute infections (Gabrilovich et al., 2012). Myeloid precursor accumulation results from this process, which compromises the ability of dendritic cells to deliver antigens and the cytotoxic protection mediated by macrophages. Initially, immature myeloid cells that have been mobilized may not have immunosuppressive effects, but they may promote inflammation and neovascularization, which aid in the growth of tumors. However, as tumors grow, the immature myeloid cells that are continuously created are exposed to various tumor-derived stimuli, which cause them to become strong suppressors of defense-related immune responses. Myeloid-derived suppressor cells (MDSCs) are diverse immature myeloid cells that suppress antitumor T-cell responses through different mechanisms. GBM can induce immunosuppression through the accumulation of MDSCs and regulatory T cells (Ostrand-Rosenberg and Sinha, 2009; Kamran et al., 2018). Understanding the complexities of glioblastoma necessitates moving from a local microenvironment viewpoint to a systemic one, in which the host macroenvironment plays a critical role in tumor formation (Sanegre et al., 2020). Numerous systemic and local variables interact intricately to influence the development of tumors and the TME. The composition of the TME and subsequent growth of tumors are significantly influenced by local variables, such as the immune response, the extracellular matrix, and adaptive angiogenesis (Obradović et al., 2019). Furthermore, a variety of systemic host variables can have a substantial impact on the response to treatment, including intestinal dysbiosis, stress-associated neurotransmitters and neurohormones, metabolic abnormalities in the tumor and host, latent infections, and surgical and physicochemical stimulation (Galluzzi and Kroemer, 2019).

## 4 Effects of endocannabinoids in the glioma microenvironment

Subcutaneous or intracranial injections of human or syngeneic glioma cells into immunodeficient mice or immunocompetent rats have demonstrated the anti-glioma effects of THC and other cannabinoid receptor agonists (Carracedo et al., 2006; López-Valero et al., 2018); these studies provide evidence of the precise role that cannabinoid receptors play in the development of gliomas. Glioblastoma cells can manipulate almost all surrounding cell types to promote tumor growth. To promote tumor growth, glioblastoma cells can, for instance, increase angiogenesis, attract astrocytes, elude macrophages and microglia, and even alter the surrounding extracellular matrix (Broekman et al., 2018). Furthermore, a substantial amount of data indicates that neural activity plays a role in regulating the course of gliomas (Gillespie and Monje, 2018). Regrettably, little research has investigated the function of cannabinoid receptors on different types of cells in the brain-tumor milieu. However, a few potential mechanisms have been proposed in the present: cannabinoids can hinder glioma angiogenesis by blocking the generation and activation of vascular endothelial growth factors and by reducing the migration and survival of vascular endothelial cells (Blázquez et al., 2004). Similarly, *in vivo*, deletion of the FAAH gene results in antiangiogenic effects (Rieck et al., 2021). Additionally, activated tumor-associated astrocytes (astrogliosis) can be manipulated by glioblastoma cells to promote the growth of tumors (O’Brien et al., 2013). As cannabinoids prevent astrogliosis in a variety of clinical contexts (Feliú et al., 2017; Espejo-Porras et al., 2019; Ruiz-Calvo et al., 2019), astrocytes linked to tumors may be rendered inactive by cannabinoid receptor interactions. Furthermore, glioma cells produce a variety of neurotransmitter receptors and connect with neurons in a synapse-like manner, which affects the formation of tumors (Venkataramani et al., 2019; Venkatesh et al., 2019). Specifically, glutamate stimulates the proliferation, migration, and survival of glioma cells via AMPA receptors (Takano et al., 2001; Ishiuchi et al., 2002). Since the primary role of CB1R is to inhibit neurotransmission (Piomelli, 2003), one possible mechanism underlying the anticancer effect of cannabis is the inhibition of glutamate release from neuron terminals. In the future, mouse models of CB1R/CB2R loss- or gain-of-function and cancer driver mutations in particular cell lineages may be used to investigate these and other theories.

**FIGURE 2:**
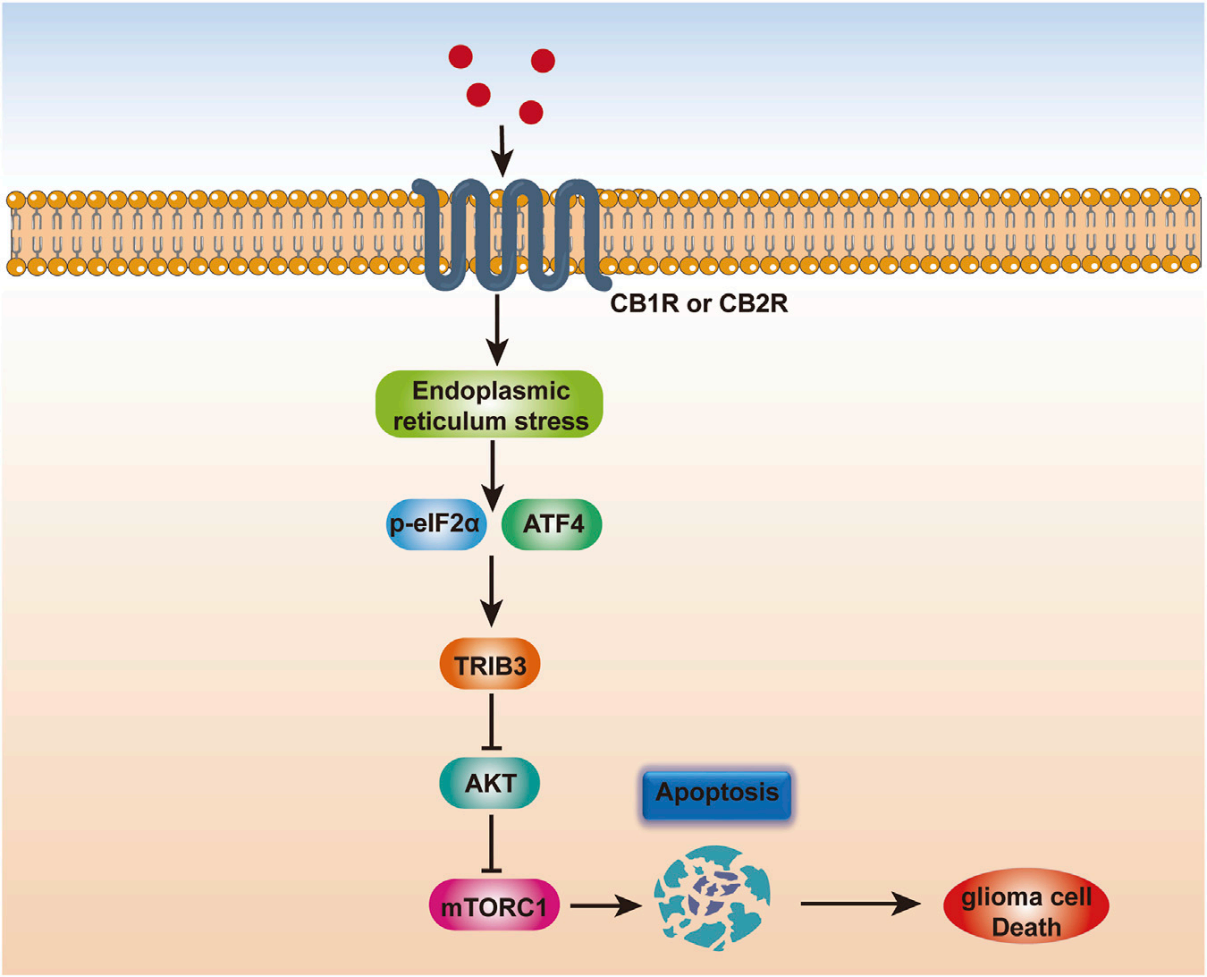
Scheme depicting the mechanism of endocannabinoid-induced glioma cell apoptosis. Endocannabinoids trigger endoplasmic reticulum stress in glioma cells, which may converge at the phosphorylation of eIF2α and the induction of ATF4. ATF4 further increases TRIB3 expression, thereby leading to inhibition of the AKT-mTORC1 axis and subsequent activation of apoptosis in glioma, thereby inducing glioma cell death.

The dysregulation of endocannabinoids and their receptor expression in the glioblastoma microenvironment during disease is thought to contribute to the development and spread of GBM (Costas-Insua and Guzmán, 2023). The effects of cannabis on the formation of GBM tumors are mediated through a variety of pathways, including those that promote cell death and inhibit angiogenesis and proliferation. Stimulation of the intrinsic apoptosis pathway by contact with a cannabinoid receptor causes an increase in intracellular ceramide, which in turn inhibits the PI3K/Akt and Raf1/MEK/ERK pathways, leading to cannabinoid-induced cell death (Ellert-Miklaszewska et al., 2013). Furthermore, a crucial signaling system that controls cell survival, proliferation, and metabolism is the PI3K/Akt/mTOR pathway. Cannabinoid-mediated activation of the CB1 and CB2 receptors inhibits the PI3K/Akt/mTOR pathway in glioblastoma cells, which reduces cell growth and promotes apoptosis and autophagy (Salazar et al., 2009). Downregulation of the expression of downstream targets such as mTOR, p70S6K, and 4EBP1, as well as the inhibition of Akt phosphorylation and activation, underlie this effect (Ciechomska et al., 2013) (Figure 2).

Oxidative stress is another factor that causes cannabinoid-induced apoptosis, as demonstrated by the increase in reactive oxygen species (ROS) production in glioma cells treated with CBD (Massi et al., 2010; Wang et al., 2021). Another significant signaling system that controls cell survival, differentiation, and proliferation is the MAPK/ERK pathway. Cannabis ligands that activate CB1 and CB2 receptors alter the MAPK/ERK pathway in glioblastoma cells, which inhibits angiogenesis and promotes apoptosis (Blázquez et al., 2003; Widmer et al., 2008). This effect is the result of downregulating the expression of downstream targets such c-fos and c-jun, as well as inhibiting ERK phosphorylation and activation. Since the JNK pathway controls both cell survival and death and is a stress-activated signaling pathway (de Los Reyes Corrales et al., 2021). When cannabis binds to CB1 and CB2 receptors, the JNK pathway is triggered in glioblastoma cells, which promotes apoptosis (Fonseca et al., 2017). Furthermore, overexpression of downstream targets such as c-jun and activation of JNK phosphorylation are the causes of this effect. To completely elucidate the molecular pathways underlying cannabis action in glioblastoma and the possibility of creating cannabinoid-based therapeutics for this fatal illness, further investigations are required (Rodriguez-Almaraz and Butowski, 2023).

Membrane phospholipid precursors are the source of endogenous AEA production. In the presynaptic region, AEA interacts with MAGL, while in the postsynaptic region, AEA interacts with FAAH. Researchers are investigating how FAAH inhibitors affect Ca2+ entry, apoptosis, and oxidative stress in human glioblastoma cell lines *in vitro*. Additional research has demonstrated that in human glioma neurons, AEA and capsaicin together induce apoptosis, oxidative stress, and Ca^2+^ buildup via the TRPV1 channel (Zhang et al., 2019). CB1 and CB2 receptors are activated by AEA; however, the presence of FAAH lowers cytosolic AEA levels. As a result, blocking FAAH causes AEA accumulation and activation of the CB1 receptor.

Another enzyme involved in the breakdown of 2-AG in presynaptic neurons is MAGL. MAGL has been linked to obesity, diabetes, and neurological diseases in addition to being observed in numerous types of cancer (Ceccarelli et al., 2016). Given that MAGL inhibition reduces the proliferation, invasion, and metastasis of cancer cells, MAGL may be a viable therapeutic target for GBM.

The Cannabis sativa plant also contains a wide range of cannabinoids. Among the many distinct types of cannabinoids, the most well-known ones are cannabidiol (CBD) and Δ9-tetrahydrocannabinol (Δ9-THC). It is believed that Δ9-THC is the psychotic cannabinoid; its connection with the CB1 receptor is responsible for many of its psychoactive effects, while its contact with the CB2 receptor is probably responsible for its immune-modulatory qualities. Conversely, CBD exhibits no psychotropic properties and a comparatively modest affinity for both CB1 and CB2 (Thomas et al., 2007). Thus, unlike △9-tetrahydrocannabinol (△9-THC), CBD is characterized by a lack of psychotropic action (Silvestro et al., 2020). CBD exhibits potent antiproliferative and pro-apoptotic properties against a wide range of cancer types in both murine tumor models and cultured cancer cell lines. Depending on the type of tumor, the anticancer processes might take many forms, such as cell-cycle arrest, induction of cell death, or multiple simultaneous mechanisms (Seltzer et al., 2020).

In GBM, CBD inhibits the PI3K/AKT survival pathway by downregulating the phosphorylation of AKT1/2 (p-AKT) and p42/44 MAPKs without effecting the total AKT and p42/44 MAPK protein levels (Solinas et al., 2013; Scott et al., 2015). Given that PTEN is increased and AKT is downregulated in glioma stem-like cells, this pathway may also be in charge of CBD-mediated autophagy in those cells (Nabissi et al., 2015). In U251, △^9^-THC and CBD together, but not separately, downregulated p42/44 MAPKs (Marcu et al., 2010). Previous studies have found that CBD treatment together with γ-irradiation led to the upregulation of active JNK1/2 and p38 MAPK, especially in U87MG cells (Ivanov et al., 2017). However, using U251 cells has demonstrated that △9-THC and CBD did not elevate JNK1/2 or p38 MAPK activity (Marcu et al., 2010). The disparity may result from genetic variations across distinct GBM cell lines.

Finally, It has been demonstrated that neurons mainly control the TME via promoting cell proliferation (Liu et al., 2011). Previous research has demonstrated that the pace of glioma cell network branching was accelerated by neural activation (Venkataramani et al., 2022). Glioma cells and neurons generate synapses, and the glioma depolarization that occurs across these synapses promotes cell proliferation (Venkatesh et al., 2019). Additionally, it was demonstrated that exposure to neuroligin-3 (NLGN3), which is secreted and degraded by a-disintegrin and metalloprotease 10 (ADAM10), promoted synaptic development. Research has demonstrated that NLGN3 affects glioma cells and neurons in a paracrine manner. The MAPK and AKT-mTOR pathways have been demonstrated to promote the formation of gliomas when activated by NLGN3 (Venkatesh et al., 2017). The Glutamate and AMPA are two other neuronal-related paracrine/autocrine singlings that have been demonstrated to promote the formation of gliomas (Ishiuchi et al., 2007). As we know, activation of CB1 and CB2 receptors by cannabinoids has been shown to inhibit the PI3K/Akt/mTOR pathway in glioblastoma cells, leading to a decrease in cell proliferation and an increase in apoptosis and autophagy (Quigley et al., 2009). Furthermore, it is now recognized that glioma cells generate neuroglial synapses—microtubes that can form synapses with neurons. The glutamatergic AMPA receptors at these synapses serve as the starting point for postsynaptic currents. These synapses’ electrical stimulation promotes the proliferation of glioma cells in turn (Venkataramani et al., 2019).

## 5 Conclusion

The current first-line treatment for glioblastoma, known as the “Stupp regime,” is consists of three steps: surgery, radiation therapy plus concurrent temozolomide, and adjuvant temozolomide (Stupp et al., 2009). While the use of additional chemotherapeutic medications for glioblastoma patients has been examined, as well as antibody or gene therapy-based approaches, but no trial conducted to date has been exceptionally successful (Wen et al., 2020). Thus, the development of novel therapeutic approaches for the treatment of glioblastoma is imperative. The main focus of anticancer medicines today is on “personalized,” molecularly targeted interventions rather than on nonspecific chemotherapy and radiation therapy. As previously mentioned, in this particular context, the growth of glioblastoma cells is effectively inhibited in animal models (mice and rats) by engaging an unambiguous molecular target (CB1R/CB2R) by a family of selective compounds (THC and other cannabinoid receptor agonists) through a defined mode of antitumor action (Rocha et al., 2014; Luís et al., 2020). However, significant knowledge gaps exist and further research to maximize the efficacy of cannabinoid receptor-targeted therapies is needed. Future studies investigating the specific signaling mechanisms of the endocannabinoid system that mediate the response of glioblastoma to immunological, mechanical, and hormonal stimuli are warranted. Furthermore, investigating the possibility of synergistic combination therapies that combine endocannabinoid-based interventions with currently used treatment methods may present a viable path toward better patient outcomes. To fully achieve the clinical promise of this unique strategy, further research must address challenges related to drug delivery, potential off-target effects, and interpatient variability in responsive to cannabinoid-based therapy. In summary, there is strong evidence supporting the application of endocannabinoids for their appealing anticancer effect against GBM. However, to investigate the effectiveness and potency of endocannabinoids, clinical testing is required. Endocannabinoids represent a potentially effective treatment approach for GBM in the future.
